# Interventions aimed at increasing the level of physical activity by including organised follow-up: a systematic review of effect

**DOI:** 10.1186/1471-2296-15-120

**Published:** 2014-06-17

**Authors:** Eva Denison, Gunn E Vist, Vigdis Underland, Rigmor C Berg

**Affiliations:** 1 Norwegian Knowledge Centre for the Health Services, Oslo, Norway

**Keywords:** Health behavior, Primary health care, Review

## Abstract

**Background:**

Organised follow-up is a common feature of several strategies at the primary health care level to promote health behaviour change, e.g. to increase physical activity. In Norway, municipal ‘healthy living’ centres run by health care personnel are established to offer counselling and organised follow-up of health behaviour change during a 12-week programme. We report the results of a systematic review commissioned by the Norwegian Directorate of Health concerning organised follow-up to improve physical activity.

**Methods:**

We searched ten electronic databases up to June 2012, reference lists of included publications, and relevant journals. Study selection and quality risk of bias assessment were carried out independently. Data were synthesised narratively due to heterogeneity of measurements of physical activity. The GRADE approach was used to assess our confidence in the effect estimate for each outcome in each comparison.

**Results:**

Fourteen randomised controlled trials from seven countries and with a total of 5,002 participants were included in the systematic review. All studies were carried out in primary care or community settings. The interventions comprised referral to supervised group physical activity (2 studies), referral to local resources with follow-up (6 studies), and self-organised physical activity with follow-up (6 studies). The narrative synthesis, comprising a total of 39 comparisons, indicated effects of self-organised physical activity with follow-up (compared to both advice and no treatment) and referral to local resources with follow-up (compared to advice) in some of the comparisons where we rated our confidence in the effect estimates as moderate. However, the results indicated no difference between intervention and control groups for the majority of comparisons. Follow-up in the studies was mainly short-term with the longest follow-up 9 months post-treatment. We rated our confidence in the effect estimates as low or very low in most comparisons, both for positive and neutral results.

**Conclusions:**

The results of this systematic review indicate considerable uncertainty concerning effects of organised follow-up during 10–14 weeks on physical activity. Major methodological problems concerning the measurement of physical activity are discussed.

**Trial registration:**

**Systematic review registration:** PROSPERO CRD42011001598.

## Background

Physical inactivity has been identified as the fourth leading risk factor for global mortality (6% of deaths globally), and it is estimated that approximately 21–25% of breast and colon cancer, 27% of diabetes, and approximately 30% of ischemic heart disease can be attributed to physical inactivity [1]. Participation in regular physical activity (PA) is known to reduce the risk of several non-communicable diseases [2].

Current guidelines on PA for adults aged 18–64 years state that each week, 150 minutes of moderate-intensity aerobic PA should be done or at least 75 minutes of vigorous-intensity aerobic PA or an equivalent combination of moderate- and vigorous-intensity activity [3]. The primary health care level is well suited to identify persons with unhealthy behaviours such as physical inactivity as 70-80% of adults in developed countries may visit a general practitioner at least once a year [4]. Several strategies to improve the ability to promote healthy behaviours of primary care patients have been reported in recent years. These include establishing ‘bridges’ between primary care practices and communities [5], ‘community health education liaisons’ [6] and ‘medical assistant referral programmes’ [7]. Strategies more specifically directed at PA include PA promotion [8], exercise referral schemes [9], and systematically integrating PA promotion into the primary care setting by means of ‘physical activity pathways’ [10]. Recent systematic reviews of PA promotion based in primary care [11] and exercise referral schemes [12] indicate small to moderate improvement of self-reported PA at 12 months’ follow-up, while only process evaluation data are available for the physical activity pathways so far [10].

In Norway, the Directorate of Health has supported the development of municipal ‘healthy lifestyle’ centres (in Norwegian, *frisklivssentraler*) since 2004. Briefly, healthy lifestyle centres are organisations at the primary health care level run by health care personnel who offer a structured, yet flexible programme for counselling and organised follow-up of behaviours that may increase risk of disease in adults [13]. One important feature of the centres is the co-ordinating function between primary health care and community resources. Presently, targeted behaviours are PA, diet, smoking, and alcohol use. Persons can be referred to a healthy lifestyle centre by health care professionals or they make contact without a referral. A programme period lasts 12 weeks, starting with a motivational health conversation which includes formulation of goals and an individual plan for the period. Several options then exist for each of the targeted behaviours. A person with a goal to increase PA may choose among individual counselling, supervised group exercise at the centre, or referral to local resources such as leisure centres or sports organisations. Follow-up during the programme period can be by individual meetings, phone, e-mail or text messages. The programme concludes with a second motivational health conversation in which goal attainment and need for another programme period is assessed. Although the focus of the programme is to prepare participants for self-organised activities, several periods with follow-up from the healthy lifestyle centre may be needed to accomplish behavioural change.

The Norwegian Directorate of Health commissioned a systematic review of the effects of organised follow-up at the primary health care/community level on behaviour that may increase risk of disease in adults (physical activity, diet, smoking, and alcohol use). The review [14] was used to inform a national guideline for municipal healthy lifestyle centres.

In the present paper we focus on the review of effects of organised follow-up on PA. Because persons can make contact with healthy lifestyle centres without a referral, the recent systematic reviews on effects of exercise referral schemes [12], and effectiveness of PA promotion based in primary care [11] would only partially answer our question, since these included studies in which participants were referred from or recruited via primary care. During the course of the review, issues related to the measurement of the primary outcome, PA, and potential consequences for data synthesis and interpretation of the results became evident. Implications of these issues will be discussed.

Thus, in this paper we aim to 1) systematically review and report the results of relevant studies concerning effects of organised follow-up on PA, and 2) discuss issues in data synthesis and interpretation of results from non-standardised reporting of PA outcomes and measurement in the included studies.

## Methods

### Inclusion criteria

#### Study design

We considered study designs in the following order: overviews of systematic reviews and systematic reviews, randomised controlled trials, cluster-randomised controlled trials, quasi-randomised controlled trials, controlled before-and-after studies, and interrupted time-series analyses.

#### Setting

Primary health care/community.

#### Population

Adults (≥18 years) with low levels of PA or increased risk of, or diagnosis of, disease for which physical activity may be a protective factor, e.g. cardiovascular disease, and type 2 diabetes.

#### Intervention

Organised follow-up over a period of 10–14 weeks, individually or in groups, given within a local organisation or by a single health professional (excluding general practitioners), starting with individual goal-setting and aiming to support increased PA.

#### Comparisons

1) Advice (with or without written information) about PA from health professionals without organised follow-up outside the office, 2) usual care, and 3) no treatment.

#### Outcomes

The primary outcome was PA behaviour, e.g. frequency, duration, intensity, achievement of pre-set goals, or indications of PA, e.g. energy expenditure, aerobic capacity.

#### Language

There were no language restrictions for the literature search. Publications in other languages than English or Scandinavian have been translated if judged relevant.

### Literature searches

We searched MEDLINE, EMBASE, Cochrane Database of Systematic Reviews, Cochrane Central Register, DARE and HTA (via Centre for Reviews and Dissemination), Cinahl, PsychINFO, Sociological Abstracts, and Social Science citation Index up to October 2011. In addition, we searched manually in a) reference lists of relevant systematic reviews identified in the electronic search, b) reference lists of included publications, c) the following journals that most commonly publish papers that could potentially match our inclusion criteria (publication dates were January 2009 – February 2012): *American Journal of Preventive Medicine, BMC Family Practice, BMC Public Health, European Journal of Public Health, Preventive Medicine, Scandinavian Journal of Primary Health Care,* and *Scandinavian Journal of Public Health*, and d) reference lists of relevant protocols identified in c). The search was updated by a search in MEDLINE in June 2012. The search strategy for the 2012 search is available in Additional file 1.

Two authors (ED and GEV, ED and VU or ED and RCB) independently screened titles and abstracts and assessed retrieved full texts against a set of pre-determined inclusion criteria. Discrepancies were solved by consensus or by a third person. There was no need to contact authors to provide additional data.

### Data extraction and analysis

The first author (ED) extracted study characteristics (study design, population, intervention(s), comparison(s), and outcome(s)) and study results (descriptive discrete or continuous data, effect measures and effect estimates) using a pre-designed data extraction form. One other author (GEV, VU or RCB) verified the extracted data against the full text articles. Risk of bias at study level was assessed according to the Cochrane Handbook [15] and independently by two authors. If consensus was not reached a third person was consulted. The GRADE approach [16] was used to rate our confidence in the effect estimate for each outcome in each comparison. The interventions were categorized according to main content, and in each category effect measures and effect estimates were described for each comparison. When several outcome measures were reported, we chose the measure(s) that best reflected total physical activity.

## Results

**Figure 1:**
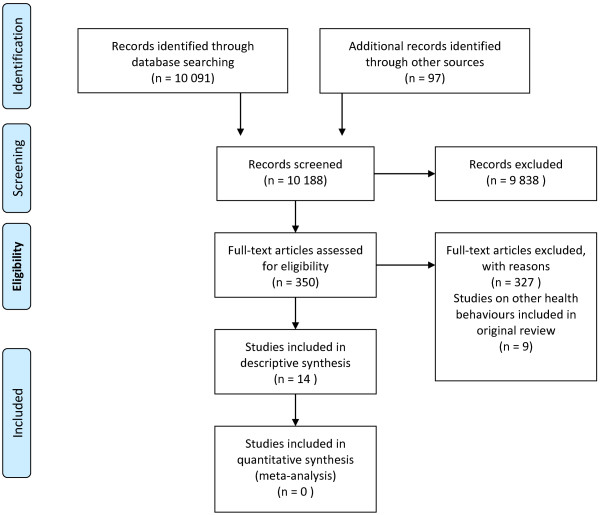
PRISMA flow diagram showing literature retrieval and study selection.

We did not find overviews of systematic reviews or systematic reviews that matched our inclusion criteria. We found 14 primary studies that met our inclusion criteria (Figure 1), all of which were randomised controlled trials [17-30]. Study characteristics and risk of bias ratings for the included studies are presented in Table 1.

**Table 1:** Study characteristics and risk of bias ratings in the included studies

**Study location**	**Design overall risk of bias**^✝^	**Participants longest follow-up**	**Intervention and duration**	**Comparison**	**Physical activity outcomes**	**Measured by**
Isaacs 2007 [17] England	RCT Unclear^3,4,5,7^	943 adults 40–74 years 3 months	Supervised PA in groups	Advice	Minutes of PA/week	Questionnaire on 7-day leisure time activities, walking, occupational activity and work in the home [48]
					Energy expenditure, kcal/kg/week	
			1) strength, fitness			
			2) walks led by instructor			
			Duration: 10 weeks			
Park 2011 [18] South Korea	RCT Unclear^1,2,3,4,5^	45 older adults ≥ 65 years Post-treatment	Supervised PA in groups	No treatment	MET-adjusted minutes of PA/week	International Physical Activity Questionnaire [49]
			Duration: 12 weeks			
Elley 2003 [19] New Zealand	Cluster-RCT Unclear^2,3,4^	878 adults 40–79 years 9 months	Referral to local resources w/follow-up	Advice	Energy expenditure, kcal/kg/week	Auckland Heart Study Physical Activity Questionnaire [50]
			Duration: 12 weeks			
Fortier 2011 [20] Canada	RCT Low^3^	120 adults 18–69 years 3 months	Referral to local resources w/follow-up	Advice	Minutes of moderate PA/day	Godin Leisure-Time Exercise Questionnaire [51]; accelerometer [52]
					PA score	
			Duration: 12 weeks			
Harrison 2004 [21] England	RCT Low^4^	545 adults ≥ 18 years 9 months	Referral to local resources w/follow-up	Advice	Number of participants who met PA goal (90 min/week)	Adapted 7-Day Physical Activity Recall Questionnaire [53]
			Duration: 12 weeks			
Stevens 1998 [22] England	RCT Unclear^2,3,4,5,7^	714 adults 45–74 years 4 months	Referral to local resources w/follow-up	Advice	Level of PA last 4 weeks	Adapted 7-Day Physical Activity Recall Questionnaire [53]
			Duration: 10 weeks			
Armit 2009 [23] Australia	RCT Low^2,4^	136 adults 50–70 years 3 months	1) Referral to local resources w/follow-up	Advice	Number of participants who met PA goal (150 min/week)	Active Australia Physical Activity Questionnaire [54]
			2) Self-organised PA w/follow-up			
			Duration: 12 weeks			
Taylor 1998 [24] England	RCT Unclear^2,3,4,5^	142 adults 40–70 years 6 months	Referral to local resources w/follow-up	No treatment	Minutes of PA/week	7-Day Physical Activity Recall Questionnaire [53]
					Energy expenditure, kcal/kg/day	
			Duration: 10 weeks			
Bjørk Petersen 2012 [25] Denmark	RCT Unclear^1,3,4^	655 adults ≥ 18 years Post-treatment	Self-organised PA w/follow-up	Advice	Minutes of PA/week	International Physical Activity Questionnaire [49]; ergometer [55,56]
					Increased level of PA	
			Duration: 12 weeks			
					Aerobic capacity	
Harland 1999 [26] England	RCT Low^2,3^	209 adults 40–64 years^§^ 9 months	Self-organised PA w/follow-up	Advice	PA score	National Fitness Survey Questionnaire [57]
					- level of PA	
			Duration: 12 weeks			
					- occasions with PA	
Baker 2008 [27] Scotland	RCT Unclear^1,3^	79 adults 18–65 years Post-treatment	Self-organised PA w/follow-up	No treatment	Number of steps/day	Pedometer [58]
			Duration: 12 weeks			
Green 2002 [28] USA	RCT Low^3^	316 adults 18–65 years 3 months	Self-organised PA w/follow-up	No treatment	PA score	Physician Assessment and Counselling for Exercise [59]
			Duration: 12 weeks			
Kirkwood 2007 [29] Scotland	RCT High^1,2,3,4,5^	34 adult women 30–50 years Post-treatment	Self-organised PA w/follow-up	No treatment	Energy expenditure, kcal/kg/day	Accelerometer [60]
			Duration: 12 weeks			
Kolt 2007 [30] New Zealand	RCT Unclear^2,3,4,5^	186 older adults ≥ 65 years 9 months	Self-organised PA w/follow-up	No treatment	Minutes of PA/week	Auckland Heart Study Physical Activity Questionnaire [50]
			Duration: 12 weeks			

Legend: PA = physical activity; ^✝^domains where risk of bias was rated as low, unclear or high at study level: ^1^sequence generation, ^2^allocation concealment, ^3^blinding of participants and personnel, ^4^blinding of outcome assessment, ^5^incomplete outcome data, ^6^selective outcome reporting, ^7^other potential threats to validity; ^§^data for Group 3 used.

### Participants and settings

Ten studies were conducted in primary health care [17,19-24,26,28,30] and four in community settings [18,25,27,29]. Twelve studies involved persons with low levels of physical activity, and two studies included persons with high blood pressure [18,24]. The studies included 5,002 participants in total, with a median of 67% female participants. Mean or median age varied between 44 and 74 years, reported in 9 studies [17-20,24,25,27,28,30]. The proportion of participants with ≥ 12 years of education varied between 27 and 75%, median 60%, reported in 9 studies [17-20,22,23,25,26,30]. The proportion of participants with white/European origin varied between 73 and 97%, median 95%, reported in 7 studies [17,19-23,28]. The proportion of persons who was offered, but declined participation in the studies varied between 38% and 86%. Demographic data were not reported for these persons.

### Interventions

The main content of the interventions was categorised as referral to supervised group PA [17,18], referral to local resources with follow-up [19-24], and self-organised PA with follow-up [23,25-30]. Total participant contact time over 10–12 weeks generally varied between one and four hours, except for the supervised group PA which varied between 20 and 36 hours. The interventions were mainly delivered by exercise specialists. Fidelity to the intervention protocol was reported in one study only [20] and ranged between 77 and 84%.

#### Referral to supervised group PA

The intervention was given 2–3 times/week for 10 weeks [17] or 12 weeks [18]. Each session lasted about an hour and involved indoor exercises to improve strength and aerobic capacity [17,18] or walks led by an instructor [17].

#### Referral to local resources with follow-up

The interventions comprised referral to an exercise specialist with follow-up 3–6 times over 12 weeks [20,23]; referral to an exercise specialist at a local centre with follow-up 3 times over 12 weeks [19], or at the end of the intervention [21,22]; referral to a local centre with subsidised access to a gym and follow-up twice over 10 weeks [24]. One of the studies [23] based the follow-up consultations on stages-of-change theory [31] and motivational interviewing [32].

#### Self-organised PA with follow-up

The interventions comprised use of pedometers and follow-up by an exercise specialist 3 times over 12 weeks [23], by e-mail 3 times over 12 weeks [25], or at the end of the intervention [27]; information about local facilities and activities and follow-up 6 times over 12 weeks [26]; self-help materials and follow-up 3 times over 12 weeks [28]; a ‘TeleWalk’ programme and follow-up 8 times over 12 weeks [30]; one consultation with specific advice to reach 60 minutes of brisk walking/day and individualised follow-up by postal mail, e-mail or phone [29]. The follow-up consultations were based on stages-of-change theory [31] in one study [28], on stages-of-change theory [31] and motivational interviewing [32,33] in three studies [23,26,30], and on the theory of planned behaviour [34] in one study [25].

### Comparisons

The interventions were compared to written or oral advice [17,20-23,25,26], usual care [19], or no treatment [18,24,27-30]. Because usual care does not exclude advice, we combined the categories written or oral advice and usual care into one category: advice. There were a total of 39 comparisons in the included studies: 9 concerning supervised group PA [17,18], 16 concerning referral to local resources with follow-up [19-24], and 14 concerning self-organised PA with follow-up [23,25-30].

### Outcomes and follow-up periods

#### Primary outcomes

Our primary outcome, physical activity, was measured in several ways in the included studies, as described in Table 1. All outcomes except minutes of moderate PA/day and energy expenditure (measured by accelerometer [20,29]) and aerobic capacity (ergometer test [25]) were self-reported by questionnaire. Data concerning psychometric properties of questionnaires were reported in one study only [28]. Harm was not reported in any of the studies.

#### Follow-up periods in the studies

The follow-up periods were described from baseline in all studies. The length of follow-up varied from immediately post-intervention to nine months post intervention (Table 1).

### Effects of interventions

Due to clinical heterogeneity of measurements of the primary outcome, data were synthesised descriptively. As described earlier, PA was operationalised in several different ways and mainly measured by different self-report questionnaires, which would make pooled effect estimates difficult to interpret.

#### Supervised group PA

The effect estimates for the 9 comparisons concerning supervised group PA are presented in Table 2. Significant differences favoring the intervention group were reported for two outcomes where we rated our confidence in the effect estimates as low [17]. For the remaining seven comparisons non-significant results were reported. We rated our confidence in these effect estimates as low for six outcomes and as very low for one outcome. About 85% of the participants were reported to adhere to all or parts of the intervention to which they were referred.

**Table 2:** Effect estimates for the comparisons concerning supervised group PA

**Study**	**Outcome**	**Participants (studies)**	**Comparison**	**Effect measure**	**Follow-up period/s**	**Effect estimate**	**Quality of the documentation**
Isaacs [17] Strength, fitness	Minutes of PA/week	305 (1)	Advice	% change from BL (95% CI)	Post-treatment	−13% (−29, 8)	Low^1,2^
		610 (1)			3 months	7% (−6, 22)	Low^1,2^
	Energy expenditure kcal/kg/week	305 (1)		% change from BL (95% CI)	Post-treatment	−7% (−24, 14)	Low^1,2^
		610 (1)			3 months	7% (−6, 23)	Low^1,2^
Isaacs [17] Walks	Minutes of PA/week	305 (1)	Advice	% change from BL (95% CI)	Post-treatment	19% (−4, 48)	Low^1,2^
		610 (1)			3 months	17% (3, 34)	Low^1,2^
	Energy expenditure kcal/kg/week	305 (1)		% change from BL (95% CI)	Post-treatment	18% (−4, 45)	Low^1,2^
		610 (1)			3 months	19% (4, 36)	Low^1,2^
Park [18]	MET-adjusted minutes of PA/week	40 (1)	No treatment	Mean diff, p-value	Post-treatment	1050, ns	Very low^1,3^

Legend: PA = physical activity; MET = metabolic equivalent of task, BL = baseline, ns = not significant; ^1^unclear risk of bias at study level, ^2^only one study, ^3^only one small study.

#### Referral to local resources with follow-up

The effect estimates for the 16 comparisons concerning referral to local resources with follow-up are presented in Table 3. Significant differences to the advantage of the intervention group were reported for four outcomes where we rated our confidence in the effect estimates as moderate for two outcomes [20,21] and as low for two outcomes [19,22]. For the remaining 12 comparisons non-significant results were reported. We rated our confidence in the effect estimates as low for six outcomes and as very low for six outcomes. Between 25% and 100% were reported to participate in all or parts of the intervention to which they were referred.

**Table 3:** Effect estimates for the comparisons concerning referral to local resources with follow-up

**Study**	**Outcome**	**Participants (studies)**	**Comparison**	**Effect measure**	**Follow-up period/s**	**Effect estimate**	**Quality of the documentation**
Elley [19]	Energy expenditure kcal/kg/week	878 (1)	Advice	Mean change diff (96% CI)	9 months	9.38 (3.96, 14.81)	Low^1,5^
Fortier [20]	Minutes of moderate PA/day	120 (1)	Advice	Mean diff, p-value	Post-treatment	−1.8, ns	Low^3^
		120 (1)			3 months	−1.6, ns	Low^3^
	PA score	120 (1)		Mean diff, p-value	Post-treatment	5.5, p = 0.01	Moderate^1^
		120 (1)			3 months	0.9, ns	Low^3^
Harrison [21]	Number of participants who met PA goal (90 min/week)	330 (1)	Advice	OR (95% CI)	3 months	1.67 (1.08, 2.60)	Moderate^1^
		312 (1)			9 months	1.49 (0.86, 2.57)	Low^3^
Stevens [22]	Level of PA last 4 weeks	314 (1)	Advice	Mean diff (95% CI)	4 months	1.52 (1.14,1.95)	Low^1,4^
Armit [23]	Number of participants who met PA goal (150 min/week)	91 (1)	Advice	OR (95% CI)	Post-treatment	2.07 (0.86, 5.02)	Low^3^
		91 (1)			3 months	1.14 (0.47, 2.76)	Low^3^
Taylor [24]	Minutes of PA/week	67 (1)	No treatment	Mean diff, p-value	1 month	66, ns	Very low^2,6^
		67 (1)			3 months	- 23, ns	Very low^2,6^
		67 (1)			6 months	- 4, ns	Very low^2,6^
	Energy expenditure kcal/kg/day	67 (1)		Mean diff, p-value	1 month	0.7, ns	Very low^2,6^
		67 (1)			3 months	0.1, ns	Very low^2,6^
		67 (1)			6 months	0.2, ns	Very low^2,6^

Legend: PA = physical activity, ns = not significant; ^1^only one study, ^2^only one small study, ^3^only one study an wide confidence interval, ^4^unclear risk of bias at study level, ^5^unclear risk of bias at study level + only 35% got the intervention, ^6^ unclear risk of bias at study level + incomplete data.

#### Self-organised PA with follow-up

The effect estimates for the 14 comparisons concerning self-organised PA with follow-up are presented in Table 4. Significant differences favoring the intervention group were reported for nine of the fourteen outcomes measured. We rated our confidence in the effect estimates as moderate for five outcomes [23,26-28], as low for three outcomes [25,30], and as very low for one outcome [29]. For the remaining five comparisons non-significant results were reported. We rated our confidence in the effect estimates as low for these outcomes. Between 66% and 77% were reported to participate in all or parts of the intervention to which they were referred.

**Table 4:** Effect estimates for the comparisons concerning self-organised PA with follow-up.

**Study**	**Outcome**	**Participants (studies)**	**Comparison**	**Effect measure**	**Follow-up period/s**	**Effect estimate**	**Quality of the documentation**
Armit [23]	Number of participants who met PA goal (150 min/week)	91 (1) 91 (1)	Advice	OR (95% CI)	Post-treatment 3 months	1.03 (0.41, 2,62) 2.39 (1.01, 5.64)	Low^2^ Moderate^1^
Bjørk Petersen [25]	Minutes of PA/week	655 (1)	Advice	Median diff, p-value	Post-treatment	120, p = 0.30	Low^1,3^
	Increased level of PA	365 (1)		% diff, p-value	Post-treatment	10.3, p < 0.01	Low^1,3^
	Aerobic capacity	655 (1)		Median diff, p-value	Post-treatment	- 0.7 p = 0.21	Low^1,3^
Harland [26]	Level of PA	166 (1) 179 (1)	Advice	% diff (95% CI)	Post-treatment 9 months	19% (6, 32) 8% (−5, 21)	Moderate^1^ Low^2^
	Occasions with PA	172 (1) 171 (1)		% diff (95% CI)	Post-treatment 9 months	16% (4, 29) 8% (−5, 20)	Moderate^1^ Low^2^
Baker [27]	Number of steps/day	79 (1)	No treatment	Mean change diff, p-value	Post-treatment	3 022, p < 0.001	Moderate^1^
Green [28]	PA score	256 (1)	No treatment	Mean diff, p-value	3 months	0.39, p = 0.049	Moderate^1^
Kirkwood [29]	Energy expenditure kcal/kg/day	37 (1)	No treatment	Mean diff (95% CI)	Post-treatment	294 (68, 520)	Very low^4,5^
Kolt [30]	Minutes of PA/week	175 (1) 165 (1)	No treatment	Mean diff, p-value	Post-treatment 9 months	48.9, p = 0.02 74.9, p = 0.05	Low^1,3^ Low^1,3^

Legend: PA = physical activity; ^1^only one study, ^2^only one study and wide confidence interval, ^3^unclear risk of bias at study level, ^4^only one small study, ^5^high risk of bias at study level.

## Discussion

The results of this systematic review show effects in favour of self-organised PA with follow-up (compared to both advice and no treatment) and referral to local resources with follow-up of PA (compared to advice) in some of the comparisons where we rated our confidence in the effect estimates as moderate. However, the results indicated no difference between intervention and control groups for the majority of comparisons. Follow-up in the studies was mainly short-term with the longest follow-up 9 months post-intervention. We rated our confidence in the effects estimates as low or very low in most comparisons, both for results in favour of the intervention and results indicating little or no difference between groups. This indicates considerable uncertainty concerning effects of organised follow-up during 10–14 weeks of PA.

We identified 14 studies including more than 5,000 participants. These studies assessed three different types of intervention; however, all evaluated the effect of organised follow-up aimed at increasing PA. Given this data material, we would have expected to arrive at clearer conclusions. The main reason for the lack of clarity and strength in the documentation may be the wide variation in outcomes combined with a lack of consensus on how to measure them. Several different constructs were used for the primary outcome, PA, such as amount of PA per week, number of persons who achieved a set goal of PA, number of steps per day, and energy expenditure. These constructs, in turn, were measured and reported in 14 different ways; mainly by self-report (see Table 1). A consequence of this is that the confidence in the effect estimates is reduced because none of them were reproduced or confirmed by others. In addition, the documentation for each outcome is based on relatively few events which in many cases lead to wide confidence intervals comprising the possibility of large benefit, no difference, and potential harm from the intervention. We believe that the large number of non-significant findings and our low confidence in the results in this review is a direct result of the lack of consensus regarding the main outcomes in PA and how to measure them.

We proposed in the protocol for the present systematic review that meta-analysis with a random-effects model be used to synthesise effect data across studies. Our decision in the course of the review to synthesise effect data descriptively was based primarily on our judgement that methodological diversity in the way the primary outcome was measured in the included studies would introduce heterogeneity that could potentially affect the results of meta-analyses. In particular, this would apply to potential systematic bias due to unknown responsiveness of the questionnaires that were used to assess effects of interventions, which may lead to exacerbation of the bias by a random-effects meta-analysis [15]. Measurement properties of 85 versions of physical activity questionnaires were assessed in a recent systematic review [35], the results showing that most questionnaires lacked information on content validity and that only a few had sufficient construct validity and reliability. Interestingly, only two of the questionnaires were found to be tested for responsiveness [35], a property that is important when evaluating intervention effects [36]. Similar results were reported for 13 physical activity questionnaires for the elderly [37].

Surely, synthesising the data descriptively will not make the problem disappear – the same uncertainty regarding bias introduced by methodological diversity in the measurement of PA persists. One possible solution to the problem of wide variation in outcomes that is increasingly discussed and applied is the establishment of “core outcome sets” in clinical trials [38,39]. A core outcome set (COS) is an agreed and standardised collection of outcomes that should be reported in all trials within a specific research area [39]. It does not preclude use of other outcomes, but defines a minimum set of outcomes that should always be measured and reported. In the case of PA, objectively measured outcomes, e.g. motion sensors like pedometers and accelerometers would preferably be included [40]. To date, the OMERACT (Outcome Measures in Rheumatology) collaboration appears to have the longest history of developing such outcome sets [41]. Briefly, OMERACT works in interactive consensus processes with three criteria for endorsement of a measure: truth, discrimination, and feasibility [41]. Guidance on Delphi techniques to arrive at consensus on core outcomes suggests the involvement of patients, clinicians, researchers, and facilitators [42]. A related issue that would preferably be incorporated in such work concerns the time points for follow-up in evaluations of interventions to increase physical activity. Study designs should reflect existing knowledge regarding the acquisition and maintenance of behaviour change.

Participants in the included studies appear to have been predominantly of European origin, well educated, and in the upper range of the adult life-span. We do not have demographic data for those who declined participation in the studies. Generalisation may therefore be limited to the above mentioned population.

The content of interventions in the included studies appears to match the options offered in healthy lifestyle centres, i.e. follow-up on self-organised PA, referral to local resources, and supervised group PA. Studies were included where the length of interventions matches one period in healthy lifestyle centres, which is the basic offer in Norway. In reality, it is more common than not that a second (or third) period is offered on the basis of the motivational health conversation that concludes the first (or second) period. It may be unrealistic to achieve stable behaviour change in 12 weeks [43] and the practice in healthy lifestyle centres to offer more than one period is probably a reflection of this. Therefore, the results of this systematic review may only correspond to the basic offer of a 12 week period in healthy lifestyle centres. During our study selection and inclusion process we identified several studies that evaluated longer interventions, e.g. six months e.g. [44] or 12 months e.g. [45]. We did not, however, come across studies that evaluated interventions that corresponded to the flexibility offered in healthy lifestyle centres.

Reported adherence to all or parts of the interventions indicated substantial variation among the studies evaluating referral to local resources (25-100%), and somewhat less variation among the studies evaluating self-organised PA with follow-up (66-77%). This makes it difficult to attempt to assess whether total participant contact time would have had any influence on the results because planned contact time does not necessarily equal actual contact time. A recent systematic review of levels and predictors of uptake and adherence to exercise referral schemes reported that levels of adherence ranged from 12% to 93% and that the pooled level across three randomised controlled trials was 43% (95% CI 32% to 54%). Substantial heterogeneity was reported, possibly reflecting differences in methods of defining adherence [12]. Our data, from a different sample of studies, seem to support the results reported by Pavey and co-workers [12].

The strengths of this systematic review include the systematic literature search and use of methods to minimise bias, e.g. independent study selection and assessment of risk of bias by several authors and according to pre-determined criteria. A limitation of the present systematic review is that potentially effective interventions using new information and communication technologies alone were not evaluated. Interventions that are Web-based e.g. [46] and mobile phone based e.g. [47] are increasingly used to support health behaviour change. Such interventions were excluded from our review because they typically lack personal motivational health conversations at the beginning and end of programmes, and personal follow-up. However, personal phone calls, e-mail or text messages are already used for follow-up during the programme period in healthy living centres and further integration of information and communication technologies may offer new possibilities in program administration.

## Conclusions

The results of this systematic review indicate that referral to local resources with follow-up and self-organised physical activity with follow up during 12 weeks may have positive effects on the amount of physical activity achieved by sedentary adults in the short and medium term. We rated our confidence in the effect estimates as low or very low in most comparisons, both for results in favour of the intervention and results indicating little or no difference between groups. This indicates considerable uncertainty concerning effects of organised follow-up during 10–14 weeks on PA. The diversity concerning both the conceptualisation and operationalisation of PA as well as the lack of documentation of responsiveness for all but a few of available questionnaires that measure PA represent major methodological problems in this research area. The adoption of “core sets” of outcomes and planning of time points for follow-up according to behaviour change theory may therefore result in considerable improvements of the internal validity of future research results.

## Competing interests

The authors declare that they have no competing interests.

## Authors’ contributions

ED participated in the design, study selection, data extraction and synthesis of the review. ED also hand searched journals and drafted the manuscript. GEV participated in the design, study selection, data extraction and synthesis of the review and helped draft the manuscript. VU participated in the study selection, data extraction, and synthesis. RCB participated in the study selection and data extraction. All authors read and approved the final manuscript.

## Pre-publication history

The pre-publication history for this paper can be accessed here:

http://www.biomedcentral.com/1471-2296/15/120/prepub

## Supplementary Material

Additional file 1 **Example of search strategy.** Search strategy for Ovid MEDLINE®. Note that the strategy comprises search terms to identify studies concerning physical activity, diet, smoking, and alcohol use. Click here for file
