# Supplementary material for: Interventions aimed at increasing the level of physical activity by including organised follow-up: a systematic review of effect
Source: BMC Fam Pract. 2014 Jun 17;15:120. doi: 10.1186/1471-2296-15-120 (PMC4075600; doi:10.1186/1471-2296-15-120)
Supplement: Additional file 1 — Example of search strategy. Search strategy for Ovid MEDLINE®. Note that the strategy comprises search terms to identify studies concerning physical activity, diet, smoking, and alcohol use. [file 1471-2296-15-120-S1.doc]

Database(s): **Ovid MEDLINE(R) In-Process & Other Non-Indexed Citations, Ovid MEDLINE(R) Daily and Ovid MEDLINE(R)** 1946 to (June 2012)
Search Strategy:

| **#** | **Searches** | **Results** |
| --- | --- | --- |
| 1 | Smoking/ | 108524 |
| 2 | "tobacco use cessation"/ or smoking cessation/ | 17888 |
| 3 | "Tobacco Use Disorder"/ | 6948 |
| 4 | (smok* or cigarette* or tobacco* or quitline*).tw. | 199865 |
| 5 | or/1-4 | 227319 |
| 6 | Alcohol Drinking/ | 46199 |
| 7 | alcohol-related disorders/ or alcohol-induced disorders/ or alcoholic intoxication/ or alcoholism/ or wernicke encephalopathy/ | 75197 |
| 8 | (alcohol* or drinking behavio?r or binge drinking).tw. | 209739 |
| 9 | or/6-8 | 238360 |
| 10 | Motor Activity/ | 67865 |
| 11 | exercise/ or running/ or jogging/ or swimming/ or walking/ | 93438 |
| 12 | Physical Exertion/ | 51316 |
| 13 | Physical Fitness/ | 19853 |
| 14 | Exercise Therapy/ | 22667 |
| 15 | ((physical* or aerobic or motor) adj2 (activit* or training)).tw. | 67673 |
| 16 | (exercis* or running or jogging or swimming or walking or walk? or pedometer*).tw. | 258848 |
| 17 | or/10-16 | 412040 |
| 18 | nutritional physiological phenomena/ or exp diet/ | 194397 |
| 19 | Feeding Behavior/ | 34390 |
| 20 | Food Habits/ | 17937 |
| 21 | exp Diet Therapy/ | 37678 |
| 22 | (nutrition* or diet* or feeding behavio?r or food habit?).tw. | 465874 |
| 23 | or/18-22 | 578164 |
| 24 | 5 or 9 or 17 or 23 | 1332056 |
| 25 | "referral and consultation"/ or remote consultation/ | 50534 |
| 26 | counseling/ or directive counseling/ | 26507 |
| 27 | feedback/ or exp feedback, psychological/ | 33049 |
| 28 | Programmed Instruction as Topic/ | 2407 |
| 29 | Telephone/ | 8369 |
| 30 | (advice* or counsel* or feedback or feed-back or guidance or recommendation* or referr* or refer or refers or telephone* or program*).tw. | 970632 |
| 31 | ((primary care based or communitybased or community-based or toolkit) adj1 intervention*).tw. | 1067 |
| 32 | Motivation/ | 44754 |
| 33 | Interview, Psychological/ | 11324 |
| 34 | 32 and 33 | 639 |
| 35 | (motivat* adj3 interview*).tw. | 1420 |
| 36 | or/25-31,34-35 | 1027631 |
| 37 | 24 and 36 | 134347 |
| 38 | Time Factors/ | 930099 |
| 39 | (("10" or "11" or "12" or "13" or "14") adj1 week*).tw. | 87051 |
| 40 | (("3" or "6" or "12") adj month*).tw. | 345037 |
| 41 | or/38-40 | 1306090 |
| 42 | 37 and 41 | 20670 |
| 43 | randomized controlled trial.pt. | 330713 |
| 44 | randomi?ed controlled trial.tw. | 33750 |
| 45 | or/43-44 | 339333 |
| 46 | 42 and 45 | 5734 |
